# Effects of refractive power on quantification using ultra‐widefield retinal imaging

**DOI:** 10.1186/s12886-021-01900-y

**Published:** 2021-03-20

**Authors:** Su-Ho Lim, Seongyong Jeong, Jang Hwan Ahn, Jano van Hemert, Min Sagong

**Affiliations:** 1 Department of Ophthalmology, Daegu Veterans Health Service Medical Center, Daegu, Korea; 2 Department of Ophthalmology, Yeungnam University College of Medicine, 170 Hyunchung-ro, Nam-gu, 42815 Daegu, Korea; 3 Yeungnam Eye Center, Yeungnam University Hospital, Daegu, Korea; 4 Optos plc, Dunfermline, United Kingdom

**Keywords:** Area measurement, Axial length, Retina, Ultra‐widefield imaging, Quantification

## Abstract

**Background:**

Ultra-widefiled (UWF) retinal images include significant distortion when they are projected onto a two-dimensional surface for viewing. Therefore, many clinical studies that require quantitative analysis of fundus images have used stereographic projection algorithm, three-dimensional fundus image was mapped to a two-dimensional stereographic plane by projecting all relevant pixels onto a plane through the equator of the eye. However, even with this impressive algorithm, refractive error itself might affect the size and quality of images theoretically. The purpose of this study is to investigate the effects of refractive power on retinal area measurements (quantification) using UWF retinal imaging (Optos California; Dunfermline, Scotland, UK).

**Methods:**

A prospective, interventional study comprised 50 healthy eyes. UWF images were acquired first without the use of a soft contact lens (CL) and then repeated with six CLs (+ 9D, +6D, +3D, -3D, -6D, and − 9D). Using stereographically projected UWF images, the optic disc was outlined by 15–17 points and quantified in metric units. We divided the subjects into three groups according to axial length: Groups A (22–24 mm), B (24–26 mm), and C (≥ 26 mm). The primary outcome was percentage change before and after use of the CLs. Secondary outcome was proportion of subjects with magnification effects, maximal changes > 10 %.

**Results:**

The study population was 6, 28, and 16 eyes in each group. Overall changes for the measured area were not significantly different in the whole study population. Group C had a larger proportion of magnification effects compared to Groups A and B (50.0 %, 0 %, and 3.6 %, *P* = 0.020). Measured area with plus lenses was significantly higher in Group C (*P* < 0.001).

**Conclusions:**

The use of CLs might affect quantification of eyes with long axial length when using UWF images. Ophthalmologists should consider refractive error when measuring area in long eyes.

## Background

Advances in ultra-widefield (UWF) imaging technology have allowed noninvasive, single images of the peripheral retina [1, 2]. Regardless of this advantage, UWF images include significant distortion when they are projected onto a two-dimensional surface for viewing [1, 3, 4]. Thus, stereoscopic projection algorithms have been employed to correct image distortion [1, 2, 5, 6]. A three-dimensional object (fundus image) was mapped to a two-dimensional stereographic plane by projecting all relevant pixels onto a plane through the equator of the eye [1, 3]. As this stereographic projection preserves the shape of a sphere, it enables the correct measurement of area based on calculations made on the sphere [1]. Therefore, many clinical studies that require quantitative analysis of fundus images have used this technology [1, 4, 7–10]. Moreover, a stereographic projection algorithm using axial length information can produce accurate and precise measurements relative to an intraocular ground truth standard [1, 2, 6].

However, even with these impressive advancements, refractive error itself might affect the size and quality of images theoretically [11]. In routine clinical practice, UWF images are obtained in subjects without refractive error correction, although refractive errors are capable of causing image distortion and magnification effects. Further, several previous studies using optical coherence tomography (OCT) have indicated that refractive errors affect the analysis of retinal nerve fiber layer (RNFL) thickness, leading to lower values in myopic eyes compared to normal eyes due to the differences in axial lengths and the magnification effects [12, 13].

There are many controversies regarding the effects of refractive errors on measurement of the retina. Certain investigations using OCT report that contact lens diopter does not significantly affect measurement of RNFL [14, 15]. On the contrary, other reports have suggested that RNFL thickness decreases as refractive power becomes more negative [12]. To date, there are no studies regarding the effects of refractive error on area measurement using UWF. In this context, many studies using stereoscopic measurement have empirically employed inclusion criteria between + 3 Diopters to − 3 or − 6 Diopters. Therefore, the purpose of this study was 1) to investigate the effects of refractive error in contact lenses (CLs) on area measurement in UWF images in healthy subjects with myopia and (2) to compare the magnification effects according to axial lengths.

## Methods

### Study subjects and baseline examinations

This is a single-center, prospective, interventional study conducted at Yeungnam University Medical Center between March 2018 and April 2018. The study protocol was approved by the Institutional Review Board of Yeungnam University Medical Center (IRB No. 2017-04-026). All participants provided signed informed consent, and the study adhered to the tenets of the Declaration of Helsinki.

The participants included healthy young subjects aged 18 years or older. Normal subjects were defined as those with no history of systemic or ocular disease or no presence of ocular surgery. The exclusion criteria were as follows: myopia < − 6 D, astigmatism > 1.5 D, intraocular pressure (IOP) > 21 mmHg, media opacity that obscured image acquisition, pathologic myopic changes of the retina such as posterior staphyloma, Lacquer crack, tessellated fundus, or myopic foveoschisis, or changes of the optic disc such as tilted disc configuration, myopic parapillary atrophy or glaucomatous changes. Media opacity was defined to be the presence of corneal scarring, corneal edema, cataract, or vitreous haze. Each participant underwent a routine ophthalmic examination, including evaluation of past medical history, best-corrected visual acuity, refractive error without pupil dilation (Auto-refracto-keratometer, HRK-7000 A; Huvitz Co., Ltd., Korea), IOP (TX-20 Full Auto Tonometers; Canon, Tokyo, Japan), and axial length (IOL Master500; Carl Zeiss Meditec, Gena, Germany).

Considering the fact that axial length and degree of baseline refractive error are capable of influencing the measurement of area in UWF images, we classified the subjects into three groups based on their corresponding axial lengths: below-average axial length group (Group A, between 22 and 24 mm), above-average axial length group (Group B, between 24 and 26 mm), and long axial length group (Group C, 26 mm or above). Further, we classified the subjects into two groups based on refractive error: mild myopia (Plano to − 3 D) and moderate myopia (− 3 D to − 6 D).

### Image acquisition, projection, and quantification

UWF imaging was performed using an Optos UWF system (Optos California; Dunfermline, Scotland, UK). UWF images were acquired first without the use of soft CL, and then the process was repeated using CLs of six different diopters (+ 9 D, + 6 D, + 3 D, − 3 D, − 6 D, and − 9 D). We employed the usage of various different powers of CL, because the use of CL could provide a refractive error correction for either astigmatism or axial length without the use of correction formula [12]. Acquired images were transformed to a stereographic projection image using proprietary software from the manufacturer [1]. With stereographically projected UWF images, two masked, trained ophthalmologists manually outlined the optic disc area by 15–17 points where the disc margin meets the blood vessel using Image J V.1.49b (US National Institutes of Health, Bethesda, Maryland).

**Fig. 1:**
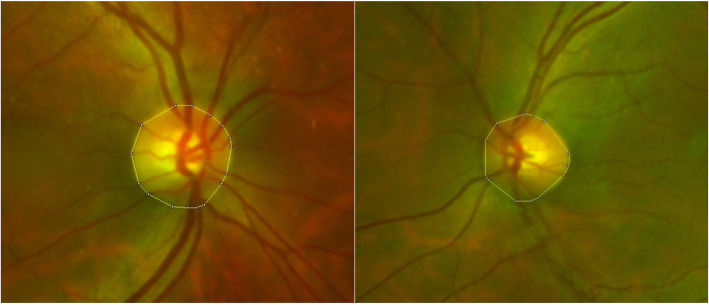
Measurement (quantification) of optic disc area. Ultrawide-field (UWF) retinal images were projected onto a flat map to preserve the peripheral aspect ratio to a best-fit 24 mm globe model. With stereographically projected UWF images, the optic disc area was outlined with 15–17 points where the disc margin meets the blood vessel. In each case, the optic disc area was measured in square millimeters (mm^2^) by summing the anatomically-corrected sizes of all pixels that comprise the disc margin

The area of the optic disc corresponding to each image was measured in square millimeters (mm^2^) by summing the anatomically-correct sizes of all pixels that comprised the disc margin (Fig. 1). To increase the reproducibility and the accuracy of measurement, we choose the optic disc as the “area of interest”, considering a method used in previous studies [1, 4, 7–10]. Two independent, masked graders performed annotations of the optic disc twice, and the average value was used for subsequent statistical analyses. These values obtained using CLs were compared with the measurements obtained from the baseline image without CL (100.0 %), and the respective percentage differences were determined. The maximal difference (%) in each case was defined as absolute value differences (% area difference) compared to those for the baseline image without CL to determine the magnification effect by refractive change in the “same eye”. The magnification group comprised subjects exhibiting maximal difference > 10 %.

### Statistical analysis

Statistical analyses were conducted using SPSS software (version 19.0; IBM Corp., Armonk, NY) and MedCalc (version 15.8; MedCalc, Inc., Ostend, Belgium). The Kolmogorov-Smirnov test was used to assess sample distribution. The differences in numerical data were analyzed using repeated measure analysis of variance (ANOVA), Kruskal Wallis test, independent t-test, and Mann-Whitney test. Categorical variables were evaluated using linear-by-linear association test and Fisher’s exact tests. Area under the curve, sensitivity, and specificity were calculated using the receiver operating characteristics (ROC) curve. A multiple comparison with Bonferroni correction was performed in cases that exhibited significant difference. Intra-grader and inter-grader agreement values were evaluated by intra-class correlation coefficient (ICC) values. Statistical significance was defined as *P* < 0.05.

## Results

### Baseline characteristics

A total of 50 eyes from 25 healthy subjects (13 male and 12 female) were included. The average age of the subjects was 25.0 ± 3.5 years; the average spherical equivalent and axial length were − 2.80 ± 1.61 D and 25.31 ± 1.07 mm, respectively. Other demographic information has been detailed in Table 1.

**Table 1:** Baseline characteristics of the study participants

Variables	Study participants (50 eyes of 25 subjects)
Age, years	25.0 ± 3.5 (Range 20–33)
Male/Female	13/12
IOP, mm Hg	15.1 ± 2.0 (Range 11–20)
Axial Length, mm	25.31 ± 1.07
Avg. Keratometry (Diopters)	42.63 ± 1.49 D
Spherical Equivalent (Diopters)	− 2.80 ± 1.61 (Range, -0.125D ~ -5.375D)

Values are presented as mean ± SD unless indicated otherwise

*IOP* intraocular pressure

### Subgroup analysis according to axial length and magnification effect

The study population was composed of 6 eyes in Group A, 28 eyes in Group B, and 16 eyes in Group C. As depicted in Table 2, the corresponding mean axial lengths were 23.13, 25.20, and 26.33 mm, respectively (Kruskal Wallis test, *P* < 0.001; post-hoc analysis using the Mann-Whitney test, Group A vs. B, *P* < 0.001; Group B vs. C, *P* < 0.001; Group A vs. C, *P* < 0.001). Subgroup analysis showed there was a significant proportion of moderate myopia subjects in Group C (linear-by-linear association test, *P* < 0.001), which also contained a larger proportion of subjects who experienced magnification effects compared to Groups A and B (50.0 %, 0 %, and 17.9 %, respectively, linear-by-linear association test, *P* = 0.006). Moreover, the absolute value of the corresponding maximal difference was also statistically significant (Kruskal Wallis test, *P* = 0.029; post-hoc analysis using Mann-Whitney test, Group A vs. B, *P* = 0.928; Group B vs. C, *P* = 0.027; Group A vs. C, *P* = 0.016).

**Table 2:** Comparison of ocular parameters according to the axial length

Variables	Group A (AL 22–24 mm) *N* = 6 eyes	Group B (AL 24–26 mm) *N* = 28 eyes	Group C (AL > 26 mm) *N* = 16 eyes	*P*-value
Age, years	24.7 ± 3.4	25.9 ± 3.6	23.8 ± 3.4	0.159^a^
IOP, mm Hg	14.2 ± 1.3	14.9 ± 2.5	15.6 ± 1.0	0.298^a^
Axial length, mm	23.13 ± 0.67	25.20 ± 0.50	26.33 ± 0.23	< 0.001^a^
Average Keratometry (D)	43.89 ± 1.39	42.79 ± 1.54	41.88 ± 1.02	0.011^a^
Spherical Equivalent (D)	-1.06 ± 0.49	-2.41 ± 1.54	-4.13 ± 0.88	< 0.001^a^
Proportion of Refractive Error				< 0.001^b^
Plano to – 3D (Mild)	6 (100.0 %)	18 (64.3 %)	2 (12.5 %)	
− 3 D to – 6 D (Moderate)	0 (0 %)	10 (35.7 %)	14 (87.5 %)	
Max. Difference (%)	3.40 ± 4.16	2.65 ± 7.9	9.36 ± 10.37	0.029^a^
Proportion of Subjects				
Max. Difference > 10 %	0/6 (0 %)	5/28 (17.9 %)	8/16 (50.0 %)	0.006^b^

Values are presented as mean ± SD unless indicated otherwise

^a^*P* value using the Kruskal Wallis test

^b^*P* value using the linear-by linear association test

*AL* axial length; *IOP* intraocular pressure

The data presented in Table 3 indicate that the magnification group contained a larger proportion of patients with long axial lengths (≥ 26 mm, 61.5 % vs. 21.6 %, linear-by-linear association test, *P* = 0.006) and moderate myopia (-3 D to – 6 D, 35.2 % vs. 84.6 %, Fisher’s exact test, *P* = 0.003), as well as greater myopic refractive error (− 4.03 D vs. − 2.37 D, Mann-Whitney test, *P* = 0.001).

**Table 3:** Comparison of ocular parameters according to magnification effects

Variables	No Magnification Group *N* = 37 eyes	Magnification Group (Max. Diff > 10 %) *N* = 13 eyes	*P*-value
Age, years	25.4 ± 3.3	23.9 ± 3.8	0.235^a^
IOP, mm Hg	15.2 ± 2.1	14.7 ± 1.7	0.381^a^
Axial Length – 24 (mm) (Mean Axial length (mm))	1.01 ± 1.05 (25.01 ± 1.05)	2.17 ± 0.45 (26.17 ± 0.45)	< 0.001^a^
Axial Length (Proportion)			
22–24 mm	6 (16.2 %)	0 (0 %)	0.006^b^
24–26 mm	23 (62.2 %)	5 (38.5 %)	
26 mm or above	8 (21.6 %)	8 (61.5 %)	
Avg. K value (D)	42.87 ± 1.51	41.94 ± 1.21	0.034^a^
Spherical Equivalent	− 2.37 ± 1.47	− 4.03 ± 1.38	0.001^a^
Refractive Error			0.003^c^
Plano to – 3D (Mild)	24 (64.8 %)	2 (15.4 %)	
− 3 D to – 6 D (Moderate)	13 (35.2 %)	11 (84.6 %)	

Values are presented as mean ± SD unless indicated otherwise

^a^*P* value using the Mann-Whitney test

^b^*P* value using the linear-by linear association test

^c^*P* value using the Fisher’s exact test

*IOP* intraocular pressure

### Receiver operating curves (ROC) and area under the ROC (AUROC)

**Fig. 2:**
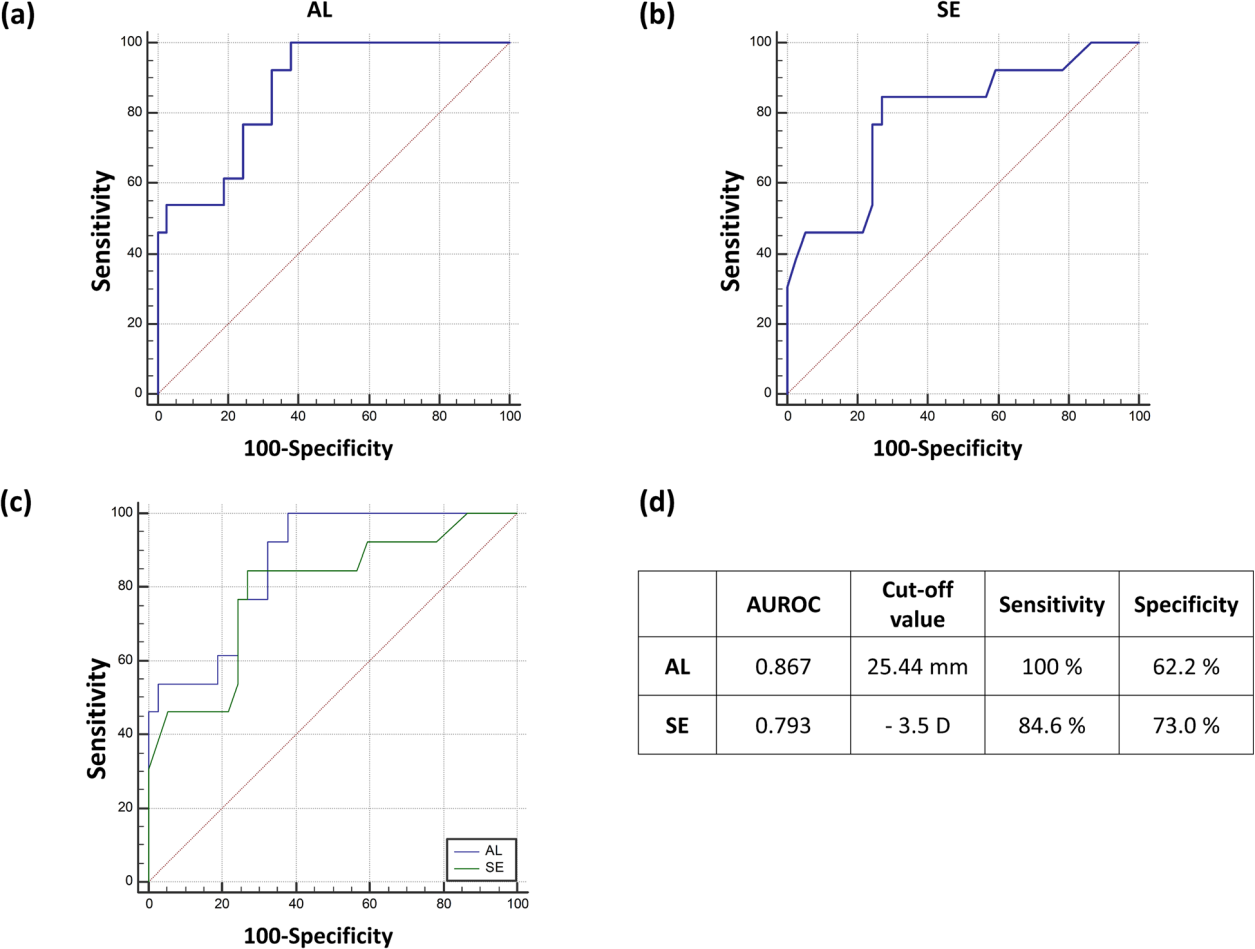
Receiver operating curves for magnification effects with cutoff values. **a** ROC curves for axial lengths, **b** ROC curves for refractive error (Spherical equivalent), **c** comparison of AUROCs (areas under ROC curves). **d** The areas under the ROC (AUROCs) and cut-off values have been provided. AL, axial length; AUROC, areas under the receiver operating curves; D, diopter; SE, spherical equivalent

Figure 2 depicts receiver operating curves for magnification effects with corresponding cutoff values. The cutoff values are 25.44 mm (sensitivity/specificity = 100 %/62.2 %) for axial length and – 3.5 Diopter (sensitivity/specificity = 84.6 %/73.0 %) for refractive error. Although areas under the ROC (AUROC) for axial length (0.867, 95 % C.I 0.741–0.946) were higher than those for spherical equivalent (0.793, 95 % C.I 0.655–0.895), there was no statistical significance. Moreover, the values with specificity > 80 % were 26.04 mm for axial length and – 4.125 Diopters for refractive error.

### Percentage changes in measured area before and after the use of the CLs

**Fig. 3:**
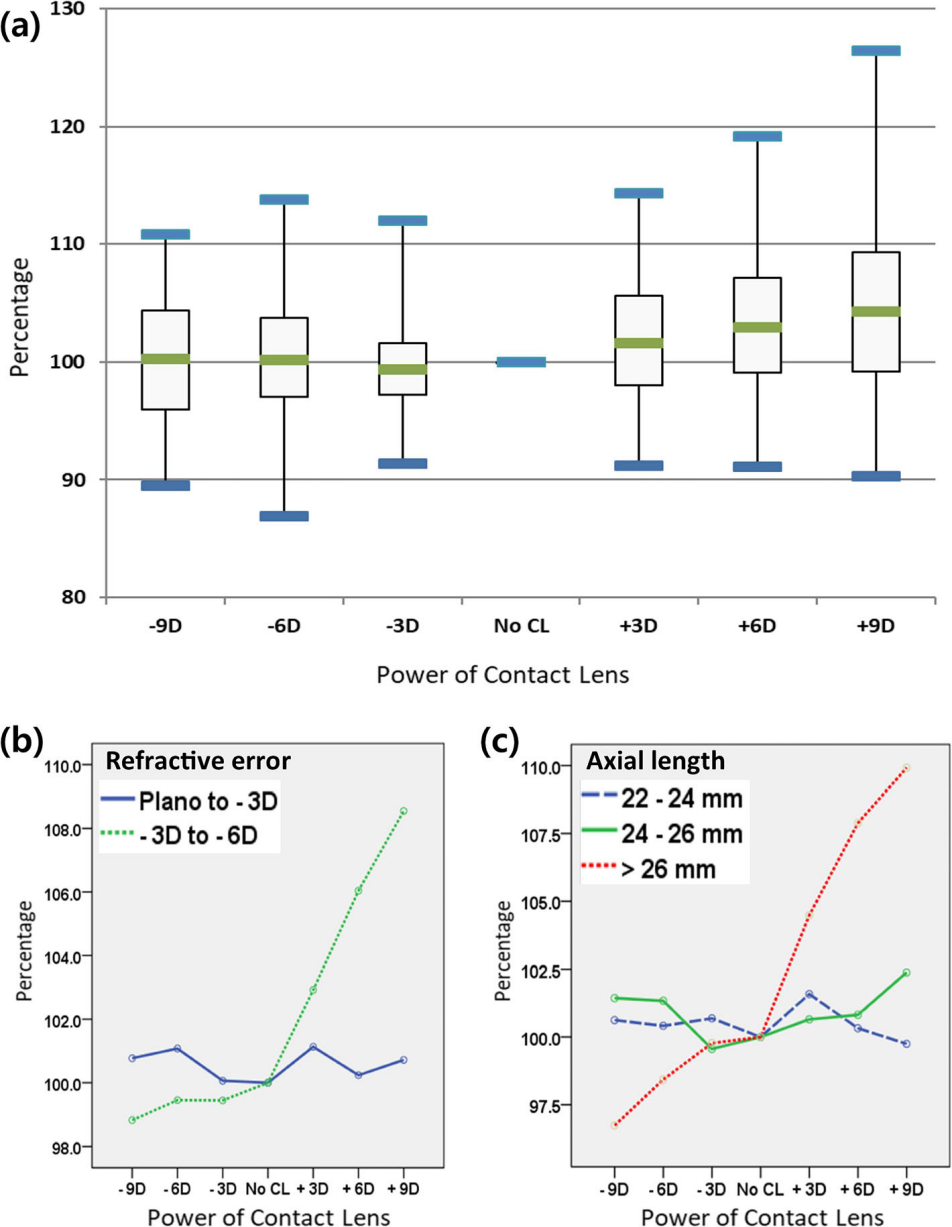
Changes in measured area with a soft contact lens compared to baseline area without a contact lens. **a** Changes in measured area with a soft contact lens compared to the measured baseline area without a contact lens in the entire population of the study. In a box-whisker plot, the boxes indicate 50 % of the values from the first to the third quartiles, and the median (green line). The upper and lower tips (blue line) represent values that are 1.5 times the interquartile range between the first and third quartiles. Comparison of percentage changes according to baseline refractive error (**b**), and axial lengths (**c**)

There was no significant difference between the CLs of different diopters (− 9 D, − 6 D, − 3 D, + 3 D, + 6 D, + 9 D) among the entire study group (Fig. 3 a). In subgroup analysis, the measured optic disc areas with the plus lenses (+ 3 D, + 6 D, + 9 D) were significantly higher than those with minus lenses in the moderate myopia group and Group C (Fig. 3b c). No adverse event associated to the application or removal of the CLs was observed.

## Discussion

In the present study, measurement of area was unaffected by refractive power in the normal axial length group (A, 22–24 mm) and the mid-axial length group (B, 24–26 mm). On the contrary, the measurement of area in the long axial length group (C, 26 mm or above) was affected by changes in refractive power.

To the best of our knowledge, no previous studies have examined the use of CL to compare area measures using UWF images. The use of CLs is capable of providing refractive error correction from either astigmatism or axial length without the use of a correction formula. In this specific population with AL ≥ 26 mm, the refractive correction achieved with a CL affected the area measurement with a statistical significance and an effect size measured in maximal difference of 9.36 ± 10.37 %. These findings are important to avoid misinterpretations or false interpretations of area measurements. Compared to measurements on UWF images using minus lenses, those using plus lenses were observed to be more affected in Group C, compared to Groups A and B.

This phenomenon is likely to be related to the myopia, with the possible hypotheses; (1) deviation from the spherical retinal model, (2) ocular magnification and (3) overestimation by grader; also a combination of these effects is possible First, we primarily consider ocular magnification effects by CL alone. A plus CL is convex, which causes light convergence. This additional lens power further focuses the scan in front of the retina, and, in particular, a positive refractive defocus error causes the scan circle to shrink in the retinal plane and become smaller in terms of the usual scan size or degrees in diameter [16, 17]. These changes could lead to an overestimation of area measurement, whereas minus CLs may similarly lead to underestimation [11]. Second, myopic eyes are elongated and may deviate from the spherical model used to perform anatomically-correct measurements.

The mean ICCs for grader 2, in particular, and between graders, were observed to be 0.942 and 0.927, respectively, suggesting good intra-grader and inter-grader reproducibility of these measurements; however, the possibility of over-estimation owing to image quality still remained [18]. With the influence of both the aforementioned effects on the estimation of the optic disc margin, ocular magnification effects are also prone to being exacerbated by the graders.

These findings are supported by previous studies using OCT. In spectral domain OCT models, signal strength and scan reliability decreased in long axial lengths [19, 20]. Similarly, increase in axial length has been confirmed to lead to ocular magnification and an underestimation of the nerve fiber layer (NFL) in SD-OCT scans without the use of a correction formula for a refractive error of less than − 4 D [13, 21, 22]. However, even correction formulas including the Litmann formula or Bennett formula are known to not be able to guarantee an accurate assessment of the optic nerve head [12, 23]. These effects may increase with increasing axial length [24].

The limitations of our study are as follows. (1) A relatively small cohort and specific sample composed of young healthy adults. (2) No subjects with hyperopic eyes or short axial lengths < 22 mm were included in the study; these subject characteristics (volunteers) reflect demographics common in the young Korean population; the prevalence of myopia in 19-year-old males in Seoul was 96.5 %, and the prevalence of high myopia (less than − 6.0 D) was 21.6 % [25, 26]. For this reason, there is a limit to extrapolating our result to hyperopic eyes. Thus, further studies are needed to clarify the magnification effects in hyperopic eyes and other ethnicities. (3) We were unable to exclude the accommodation effect; CL-induced refractive changes might exceed physiologic accommodation. (4) Myopic eyes, which are often elongated, can deviate from a spherical shape assumed for quantification. Despite these limitations, our models have certain advantages. This is the first study to analyze the effect of refractive error on the area measured after correcting the peripheral distortion by the stereographic method. In addition, the use of CL could provide a refractive error correction for either astigmatism or axial length without the use of a correction formula, such as the Littmann or the Bennett formulae.

## Conclusions

To the best of our knowledge, this is the first study to investigate the effect of refractive error on area measurement in UWF images. Our study demonstrated that refractive errors might affect area measurement accuracy in subjects with long axial length. Although the majority of the subjects exhibited no definite magnification effects, it was concluded that careful correction or consideration is required for area measurement in eyes with long axial lengths.

## Data Availability

The datasets generated during and/or analyzed during the current study are available from the corresponding author on reasonable request.

## Abbreviations

UWF: Ultra-widefield
OCT: Optical coherence tomography
RNFL: Retinal nerve fiber layer
D: Diopter
CL: Contact lens
IOP: Intraocular pressure
ROC: Receiver operating characteristics
